# Supplementary material for: Interplay between tie strength and neighbourhood topology in complex networks
Source: Sci Rep. 2024 Apr 3;14:7811. doi: 10.1038/s41598-024-58357-4 (PMC10987512; doi:10.1038/s41598-024-58357-4)
Supplement: Supplementary file 1 — Supplementary Information. [file 41598_2024_58357_MOESM1_ESM.pdf]

Supplementary Information - Interplay between Tie Strength and Neighbourhood Topology in Complex Networks

Maciej J. Mrowinski[1,*], Kamil P. Orzechowski[1], Agata Fronczak[1], and Piotr Fronczak[1]

[1]Warsaw University of Technology, Faculty of Physics, ul. Koszykowa 75, 00-662 Warsaw, Poland
[*]maciej.mrowinski@pw.edu.pl

Supplementary figures

Correlation profiles that were not included in the main text can be found in Figs. S1-S5. Additionally, Fig. S6 and Fig. S7 show the relation (consistent with Granovetter's theory) between the asymmetric overlap Q and the original asymmetric weight V of directed links for Twitter and the network of flights. Both networks are directed, and by original weights, we mean the weights of directed edges in the networks before symmetrisation (cf. the main text, where these weights of directed edges were symmetrised via Eq. 20 and then desymmetrised using Eq. 12). We do not provide any comparisons with the null model here, because, much like in the case of symmetric weights (see panels a) and b) in Figs. S1-S5), the shuffling of original weights destroys the correlation between Q and V.

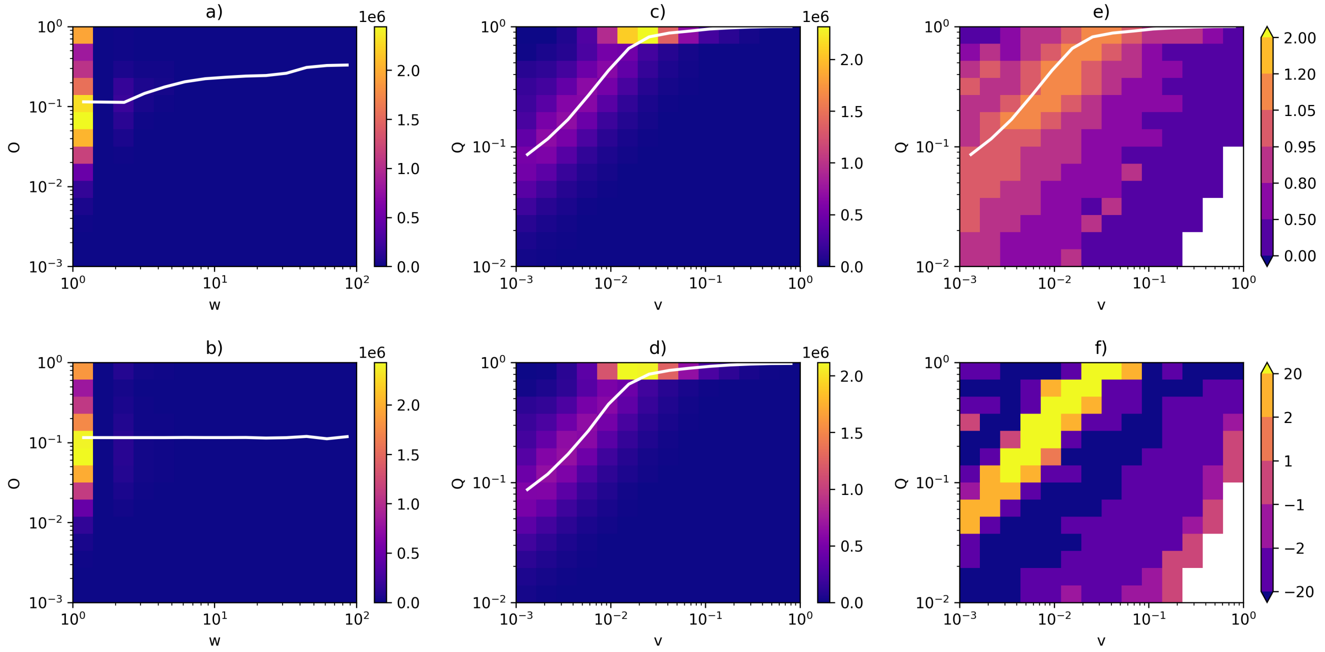

Figure S1. Correlation profiles for Actor Movies. a) Heatmap for the actual network - symmetric weights. b) Heatmap for the null model (randomised network) - symmetric weights. c) Heatmap for the actual network - asymmetric weights. d) Heatmap for the null model (randomised network) - asymmetric weights. e) Correlation profile (R). f) Z-score (Z). The white lines in a) and b) correspond to the average O as a function of w, on c) and d) - the average Q as a function of v. The line in panel e) is the same as in c).

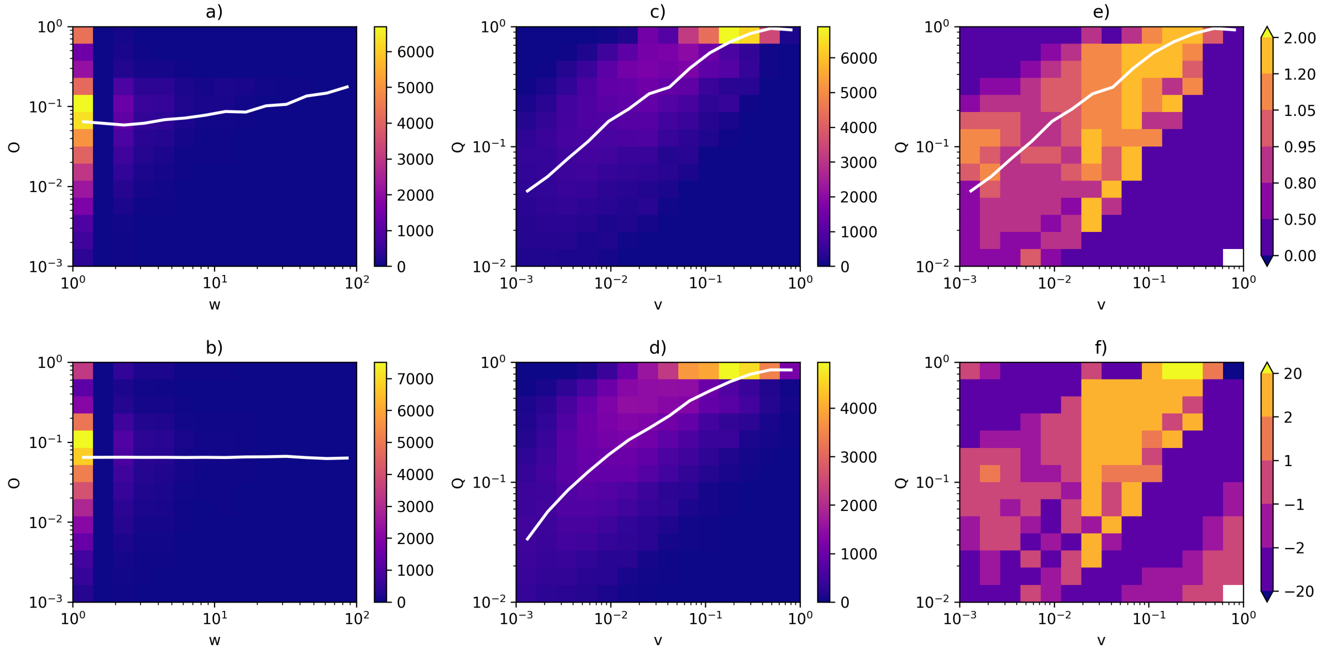

Figure S2. Correlation profiles for Record Labels. a) Heatmap for the actual network - symmetric weights. b) Heatmap for the null model (randomised network) - symmetric weights. c) Heatmap for the actual network - asymmetric weights. d) Heatmap for the null model (randomised network) - asymmetric weights. e) Correlation profile (R). f) Z-score (Z). The white lines in a) and b) correspond to the average O as a function of w, on c) and d) - the average Q as a function of v. The line in panel e) is the same as in c).

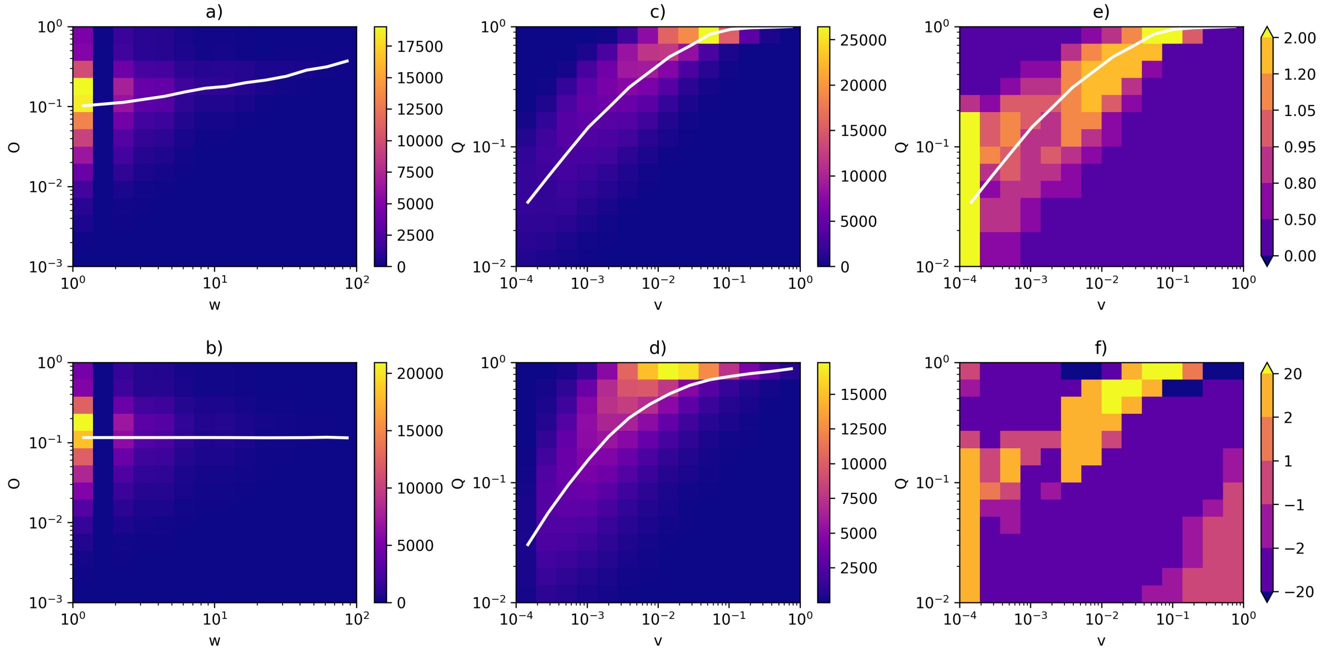

Figure S3. Correlation profiles for Marvel. a) Heatmap for the actual network - symmetric weights. b) Heatmap for the null model (randomised network) - symmetric weights. c) Heatmap for the actual network - asymmetric weights. d) Heatmap for the null model (randomised network) - asymmetric weights. e) Correlation profile (R). f) Z-score (Z). The white lines in a) and b) correspond to the average O as a function of w, on c) and d) - the average Q as a function of v. The line in panel e) is the same as in c).

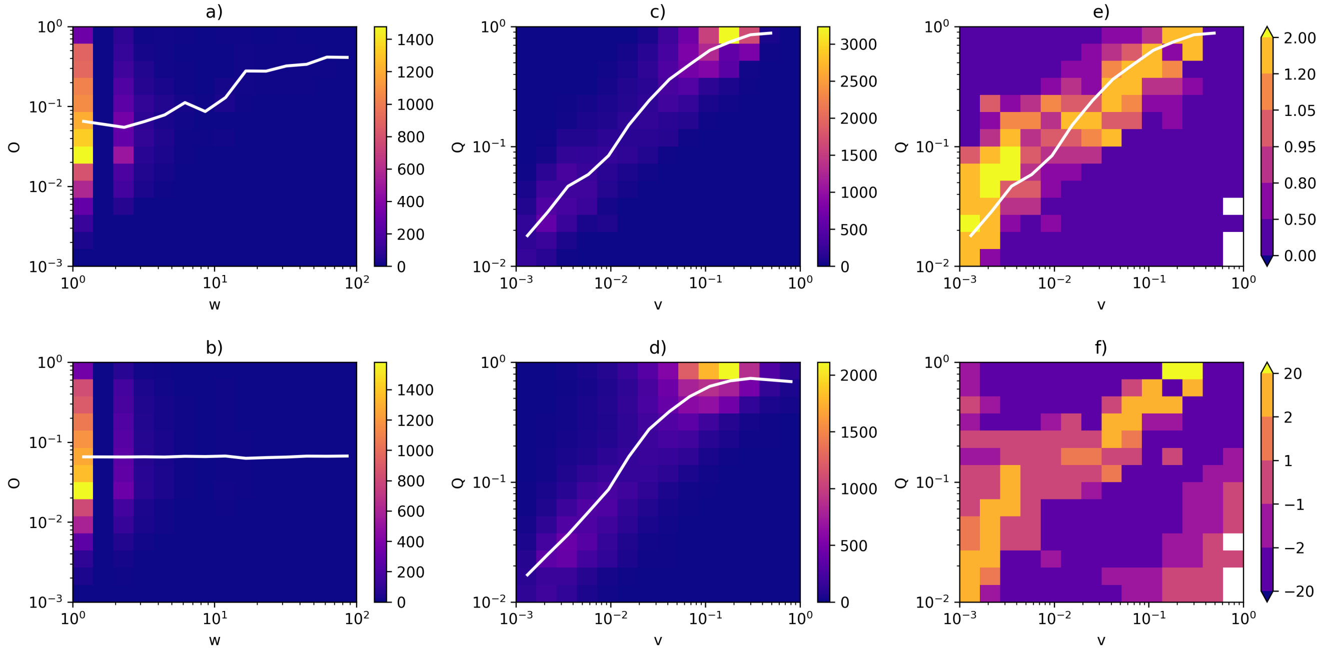

Figure S4. Correlation profiles for Metabolic Network. a) Heatmap for the actual network - symmetric weights. b) Heatmap for the null model (randomised network) - symmetric weights. c) Heatmap for the actual network - asymmetric weights. d) Heatmap for the null model (randomised network) - asymmetric weights. e) Correlation profile (R). f) Z-score (Z). The white lines in a) and b) correspond to the average O as a function of w, on c) and d) - the average Q as a function of v. The line in panel e) is the same as in c).

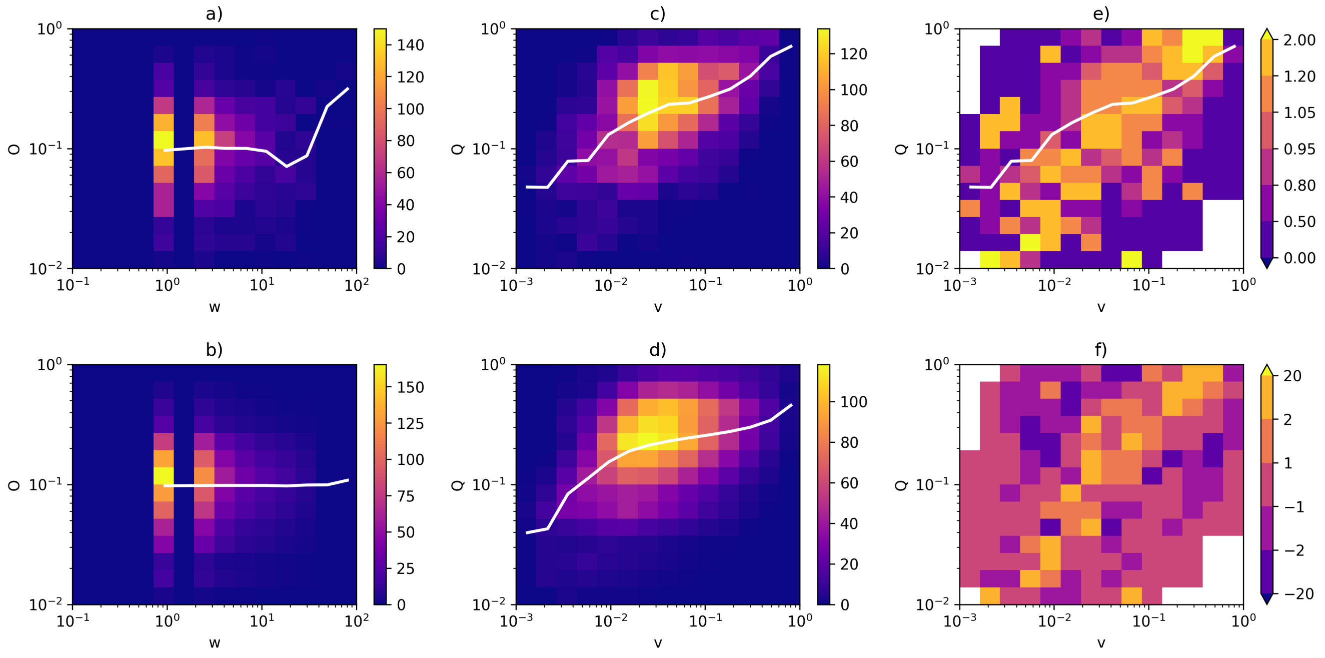

Figure S5. Correlation profiles for Caenorhabditis Elegans. a) Heatmap for the actual network - symmetric weights. b) Heatmap for the null model (randomised network) - symmetric weights. c) Heatmap for the actual network - asymmetric weights. d) Heatmap for the null model (randomised network) - asymmetric weights. e) Correlation profile (R). f) Z-score (Z). The white lines in a) and b) correspond to the average O as a function of w, on c) and d) - the average Q as a function of v. The line in panel e) is the same as in c).

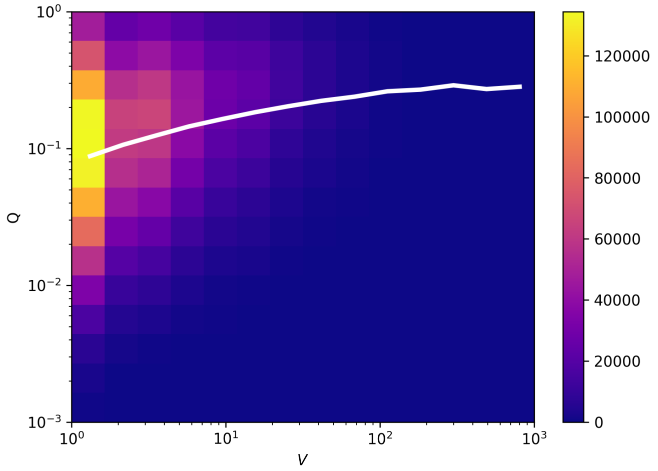

Figure S6. Heatmap of Q as a function of V (original asymmetric edge weight) for Twitter. The white line corresponds to the average value.).

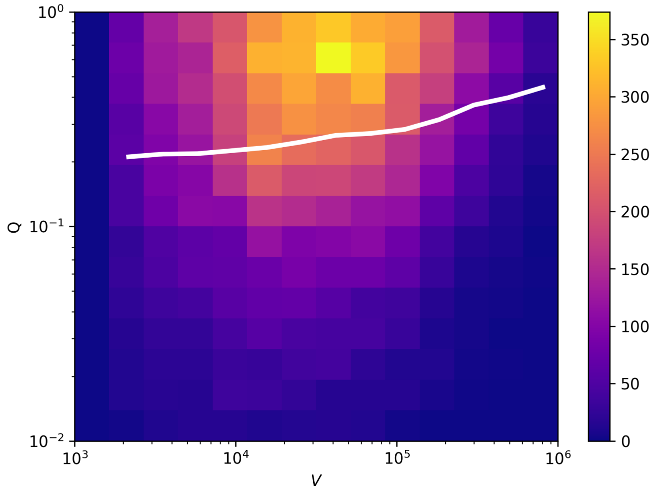

Figure S7. Heatmap of Q as a function of V (original asymmetric edge weight) for the network of flights. The white line corresponds to the average value.).
